# Rough operators: sphingomyelinase inhibitors spike NK cells to kill cancer

**DOI:** 10.1038/s41392-023-01550-0

**Published:** 2023-08-21

**Authors:** Mattias Carlsten, Yenan T. Bryceson

**Affiliations:** 1 Center for Hematology and Regenerative Medicine, Department of Medicine Huddinge, Karolinska Institute, 14157 Stockholm, Sweden; 2 Center for Cell Therapy and Allogeneic Stem Cell Transplantation, Karolinska Comprehensive Cancer Center, Karolinska University Hospital, 17176 Stockholm, Sweden; 3 Division of Clinical Immunology and Transfusion Medicine, Karolinska University Hospital, 17176 Stockholm, Sweden; 4 Broegelmann Research Laboratory, Department of Clinical Sciences, University of Bergen, 5030 Bergen, Norway

**Keywords:** Tumour immunology, Innate immune cells

In a recent study published in *Nature Immunology*, Zheng et al. discovered that intratumoral NK cells were smooth and rounded as opposed to the typical vili-rich, rougher surface of normal NK cells.^1^ This phenotype was linked to a specific deficit in membrane sphingomyelin lipids that impaired tumor cell killing. Importantly, results suggested that NK cell-mediated immunosurveillance of cancer can be rescued by blocking enzymes that degrade sphingomyelin, uncovering a potential novel ‘metabolic immune checkpoint’ for which pharmacological inhibition holds promise for cancer therapy.

NK cells are generally considered a subset of innate lymphoid cells that mediate cellular cytotoxicity. Unlike T cells that express rearranged, clonally distributed, and antigen-specific receptors, NK cells integrate signals from a multitude of germline-encoded activating and inhibitory receptors to identify infected and malignant cells. NK cells can also recognize antibody-coated target cells via low-affinity Fc-binding receptors. Despite using distinct strategies for target cell recognition, NK cells, and CD8^+^ T cells share activating signaling pathways and mechanisms for secretion of cytotoxic granules required for target cell killing. Besides blood, NK cells are abundant in liver, lung, and endometrial tissues. Ample evidence supports their role in immunosurveillance of tumors.

Advances in cancer immunotherapy have placed cytotoxic CD8^+^ T and, more recently, NK cells in the limelight. Blockade of inhibitory receptors expressed by these cells can unleash strong anti-tumor responses, potentially leading to remission of cancer. Regrettably, responses to such checkpoint blockade are only durable in a minor fraction of patients. In addition, adoptive infusion of engineered T cells as well as NK cells expressing chimeric antigen receptors have been remarkably successful against several hematological malignancies. Although tumor-infiltrating lymphocyte and T cell receptor-based therapies have highlighted the potential, adoptive cell transfer trials have largely failed against solid tumors. Multiple factors spanning from poor tumor-infiltration of cytotoxic lymphocytes to complex immunoregulatory and metabolic processes within the tumor microenvironment (TME) can explain immune evasion and insufficient responses. Several studies have documented tumor-induced suppression of CD8^+^ T cell and NK cell cytotoxicity, e.g. via loss of key signaling proteins in lymphocyte-rich tumors.

**Fig. 1:**
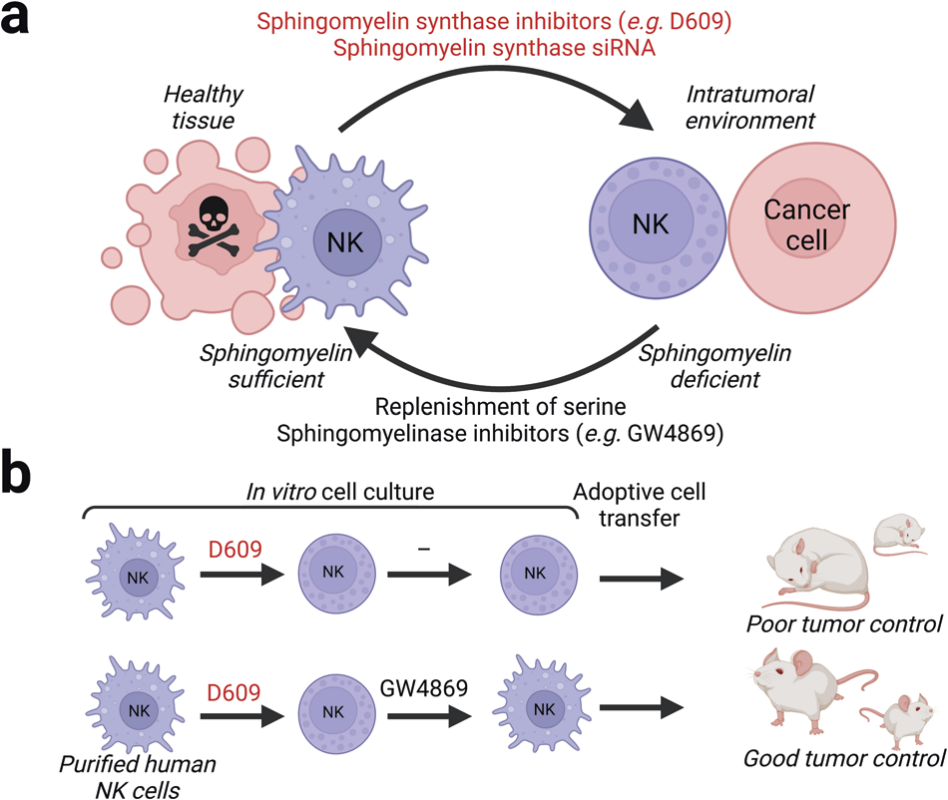
Sphingomyelin metabolism modulates NK cell-mediated control of liver cancer. (**a**) Relative to NK cells outside tumors (left), intratumoral NK cells (right) lacked membrane protrusions and failed to form tight conjugates required for tumor cell killing. This smooth, rounded NK cell phenotype was found inside liver tumors and could be recapitulated in NK cells from healthy volunteers by sphingomyelin synthase inhibition, either by pharmacological compounds or with *SGMS1* siRNA. Conversely, this phenotype could be reverted by supplementation of the sphingomyelin synthase substrate serine or by pharmacological inhibitors of sphingomyelinases. (**b**) Human liver cancer cell xenografted NOD-*Prkdcscid IL2rgem1*/Smoc mice demonstrated reduced tumor sizes when infused with human sphingomyelin-restored NK cells (GW4869-mediated restoration of D609-induced sphingomyelin deficient NK cells) compared to non-restored control NK cells (D609-treatment alone). This effect was further enhanced by anti-TIM3 checkpoint antibody blockade. The figure was prepared with BioRender

In their study, Zheng and Hou et al. addressed this issue from a new perspective comparing the ultrastructure of human NK cells from blood and tumors of the liver by electron microscopy.^1^ Strikingly, they found that intratumoral NK cells from patients with advanced stage liver cancer lacked membranous protrusions, as opposed to NK cells from non-tumor areas of the same livers or from the blood of healthy volunteers (Fig. 1). The lack of membrane protrusions corresponded to a deficit in target cell binding and killing of prototypic liver cancer cells. Next, the authors examined NK cells by advanced mass spectrometry using micropipettes capturing membrane or intracellular contents. Their most significant finding was reduced levels of multiple sphingomyelins in intratumoral NK cells. Sphingomyelin is a cell membrane lipid component that is synthesized by sphingomyelin synthase in a serine-dependent manner and catabolized by different sphingomyelinases to produce ceramide. The membrane composition of sphingomyelin can alter receptor clustering and signaling as well as membrane dynamics. In their experiments, interfering with sphingomyelin synthase activity through pharmacological inhibition or siRNA interference smoothened the NK cell surface. The authors elegantly demonstrated that intratumoral NK cells were deficient in serine, with serine supplementation to cultures resulting in reformation of the membrane protrusions. Pharmacological inhibition of acid or neutral sphingomyelinase rapidly restored membrane protrusions on NK cells. Of therapeutic relevance, such inhibition could reduce tumor sizes in a xenogeneic model of human liver cancer. The anti-tumor responses promoted by sphingomyelinase inhibition acted synergistically with checkpoint blockade. These results thus define sphingomyelinases as a potential novel ‘metabolic immune checkpoint’ and provide a rational for pharmacological modulation of sphingomyelin levels in liver cancer and potentially many other tumor types to enhance NK cell-mediated anti-tumor immunity.

Activation of acid sphingomyelinase activity has been reported down-stream of several lymphocyte co-stimulatory receptors, including CD28, LFA-1, and CD161. Nonetheless, the cellular consequences of sphingomyelinase activity in lymphocytes remain unclear. Some reports have provided evidence for ceramide inducing cellular apoptosis whereas others have implicated ceramide in promoting signals for cellular growth and survival.^2^ Notably, CD8^+^ T cells from acid sphingomyelinase deficient patients as well as knock-out mice have previously been reported to display defective killing of target cells and impaired control of viral infections,^3^ leading the authors to generally conclude that acid sphingomyelinase is required for effective secretion of cytotoxic granule content and lymphocyte-mediated target cell killing. In melanoma, expression of acid sphingomyelinase decreases with tumor progression in both humans and mice.^4^ In a mouse melanoma model, silencing of acid sphingomyelinase in tumor cells increased tumor burden.^4^ Conversely, supporting some of the findings by Zheng et al.^1^, in a murine model of lung adenocarcinoma, acid sphingomyelinase deficiency reduced tumor development and was associated with increased cytotoxic T cell-mediated anti-tumor responses.^5^ Furthermore, elevated acid sphingomyelinase activity promoted T cell apoptosis, whereas pharmacological inhibition of acid sphingomyelinase rescued CD8^+^ T cell responses in this model. Combined, these somewhat opposing observations may indicate that sphingomyelin and ceramide levels need to be finely balanced for optimal NK cell or CD8^+^ T cell-mediated immunosurveillance of cancer. It is not clear at what stage of cancer sphingomyelin composition is dysregulated and if the major causes are serine deficiency, reduced sphingomyelin synthase, or increased sphingomyelinase activity. It is possible that these mechanisms and effects may differ between different tissues and cancer types.

In conclusion, the study by Zheng and Hou et al. highlights a critical pathway for lymphocyte anti-tumor immunity. Their development of advanced methods for studying the membrane composition of individual cells will facilitate further experimental characterization of how the TME can modulate immune cell function. Their findings warrant further in vivo investigations of sphingomyelinase inhibitors in different cancer models to confirm the efficacy and possibly extend the indication for metabolic checkpoint inhibition to promote cytotoxic lymphocyte-mediated anti-tumor immunity. Future studies also need to address how current and future anti-cancer therapies can be integrated with modulation of sphingomyelin levels for optimal NK cell function within the tumor.
